# Anatomy of a range contraction: Flow–phenology mismatches threaten salmonid fishes near their trailing edge

**DOI:** 10.1073/pnas.2415670122

**Published:** 2025-03-31

**Authors:** Stephanie M. Carlson, Kasey C. Pregler, Mariska Obedzinski, Sean P. Gallagher, Suzanne J. Rhoades, Cleo Woelfle-Hazard, Nathan Queener, Sally E. Thompson, Mary E. Power

**Affiliations:** ^a^Department of Environmental Science, Policy, & Management, University of California, Berkeley, CA 94720; ^b^California Sea Grant, Santa Rosa, CA 95403; ^c^California Department of Fish and Wildlife, Fort Bragg, CA 95437; ^d^Mattole Salmon Group, Petrolia, CA 95558; ^e^Department of Civil, Environmental, and Mining Engineering, University of Western Australia, Perth, WA 6009, Australia; ^f^Centre for Water and Spatial Science, University of Western Australia, Perth, WA 6009, Australia; ^g^Department of Integrative Biology, University of California, Berkeley, CA 94720

**Keywords:** drought, extreme event, life history, migration, species redistribution

## Abstract

Climate change is redistributing life on Earth, with profound impacts for ecosystems and human well-being. While repeat surveys separated by multidecadal intervals can determine whether observed shifts are in the expected direction (e.g., poleward or upslope due to climate change), they do not reveal their mechanisms or time scales: whether they were gradual responses to environmental trends or punctuated responses to disturbance events. Here, we document population reductions and temporary range contractions at multiple sites resulting from drought for three Pacific salmonids at their ranges’ trailing edge. During California’s 2012 to 2016 historic multiyear drought, the 2013 to 2014 winter stood apart because rainfall was both reduced and delayed. Extremely low river flows during the breeding season (“flow–phenology mismatch”) reduced or precluded access to breeding habitat. While Chinook (*Oncorhynchus tshawytscha)* experienced a down-river range shift, entire cohorts failed in individual tributaries (steelhead trout, *O. mykiss*) and in entire watersheds (coho salmon, *O. kisutch)*. Salmonids returned to impacted sites in subsequent years, rescued by reserves in the ocean, life history diversity, and, in one case, a conservation broodstock program. Large population losses can, however, leave trailing-edge populations vulnerable to extinction due to demographic stochasticity, making permanent range contraction more likely. When only a few large storms occur during high flow season, the timing of particular storms plays an outsized role in determining which migratory fish species are able to access their riverine breeding grounds and persist.

## Significance

Learning how climate change alters distributions of organisms over the Earth is essential for predictive understanding. Do local populations dwindle as conditions grow more stressful, or are they abruptly extirpated? We need surveys with more spatial and especially temporal resolution to learn which climate-related factors threaten populations, and how various species, subspecies, and life history stages can respond. Here, we show in detail how a historically severe drought blocked access to critical breeding and rearing habitats for three native salmonids in rivers along the California North Coast. Different salmonids coped, not always successfully, by altering breeding timing or location. With site-specific, mechanistic understanding of hydroclimatic challenges to organisms, we can anticipate and mitigate these threats to valued natural populations.

Climate change is redistributing life on Earth. Distributional shifts along elevational, latitudinal, or depth gradients consistent with climate change have been widely documented around the globe (1–3), with profound consequences for ecosystems and human well-being (4). Inference of distributional shifts is derived from comparisons of historical and contemporary distribution data over decadal (e.g., ref. 5) to centennial (e.g., ref. 6) time scales. Shifts based on a few observation periods at the start and at the end of a time series can be correlated with general trends in climate but cannot reveal how or why distributions shifted. There is a growing interest in understanding the mechanisms underlying range shifts to increase our ability to anticipate and mitigate threats caused by species redistribution (e.g., refs. 7 and 8). While the current paradigm assumes that distributions often shift gradually in response to climate trends, a handful of examples have shown the importance of event-driven distributional shifts (e.g., refs. 9 and 10). For example, the range of a habitat-forming seaweed contracted by 5% at its warm edge due to one extreme heat event (11). Extreme climate events are increasing in severity and magnitude (12), suggesting that such event-driven distributional shifts may become more common in the future.

Phenology is a key life history trait that influences responses to climate change (e.g., ref. 13), including species range expansions (e.g., ref. 7)— in both sessile organisms like germinated plants (14) and in highly dispersive taxa like boreal Lepidopterans. In their review of 20 y of distributional data for 289 Finnish Lepidopterans, Hällfors et al. (15) concluded that dispersive species that are also shifting their phenologies are more likely to be winners under climate change. Environment-phenology mismatches caused by extreme events can also drive sudden reductions in populations at the trailing edges of their species’ biogeographic ranges.

Many riverine organisms show life history adaptations to the timing of high and low flow events (16), suggesting that altered timing of key flow events can lead to “flow–phenology mismatches.” For example, the onset of rains often cues the upriver migrations of breeding fishes [e.g., the great piracema migration in South America (17, 18)]. Similarly, in streams with low summer water levels, the onset of fall rains and corresponding elevated river flows allow upriver migrating Pacific salmon and trout to access upstream breeding habitats (19–23). We posit that when there are only a few storms during the breeding season, the timing of storms will determine which fish species are able to access their breeding grounds at the typical time. The timing and sequence of storms is particularly important, as access to breeding grounds may require travel to and passage over sequential barriers, such as shallow gravel bars in lowland mainstem rivers and natural waterfalls and stepped tributary junctions further upstream near headwaters. Here, we document multisite and multispecies impacts on salmonid populations of a single extreme hydroclimatic event: reduced and delayed rainfall during a historically severe drought in California. The 2012 to 2016 California drought may be the most severe in the region in over 21,000 y, based on analyses of tree rings (24, 25), and the winter of 2013 to 14 stood apart from the other drought years because of an abnormally late transition from the dry season to the wet season due to late rains.

Using long-term river flow records from northern California, we put the 2013 to 14 winter in context—both temporally within the long-term record and spatially along the California north coast. Adult salmon and trout enter California’s coastal rivers to breed during the wet winter, when elevated river flows allow fish to access breeding habitats. We compared the magnitude (mean flow during the breeding season) and timing (winter onset) of winter flows from the 2013 to 14 winter to the long-term record (40 recent water years from 1983 to 2022) for each of 13 coastal watersheds from Marin to Humboldt counties (*SI Appendix*, Fig. S1 and Table S1). We ranked the mean flows (lower ranks correspond with drier conditions during the breeding season) and winter onset across years (higher ranks correspond with more delayed onsets). We then combined observations from multiple monitoring efforts to illustrate the consequences of extremely delayed rains for three species of salmon and trout near their trailing range edge. While sampling designs differed among the monitoring efforts, they fell into two general categories: monitoring of adults on the breeding grounds and/or monitoring of newly hatched juvenile fish (an indication of successful breeding) (*SI Appendix*, Table S2). The adult monitoring data allowed us to explore the timing and location of breeding, while juvenile monitoring data revealed patterns of juvenile production and cohort strength.

## Results

### Flow Was Reduced and Delayed in 2013-14 Relative to the Long-Term Record.

**Fig. 1.**
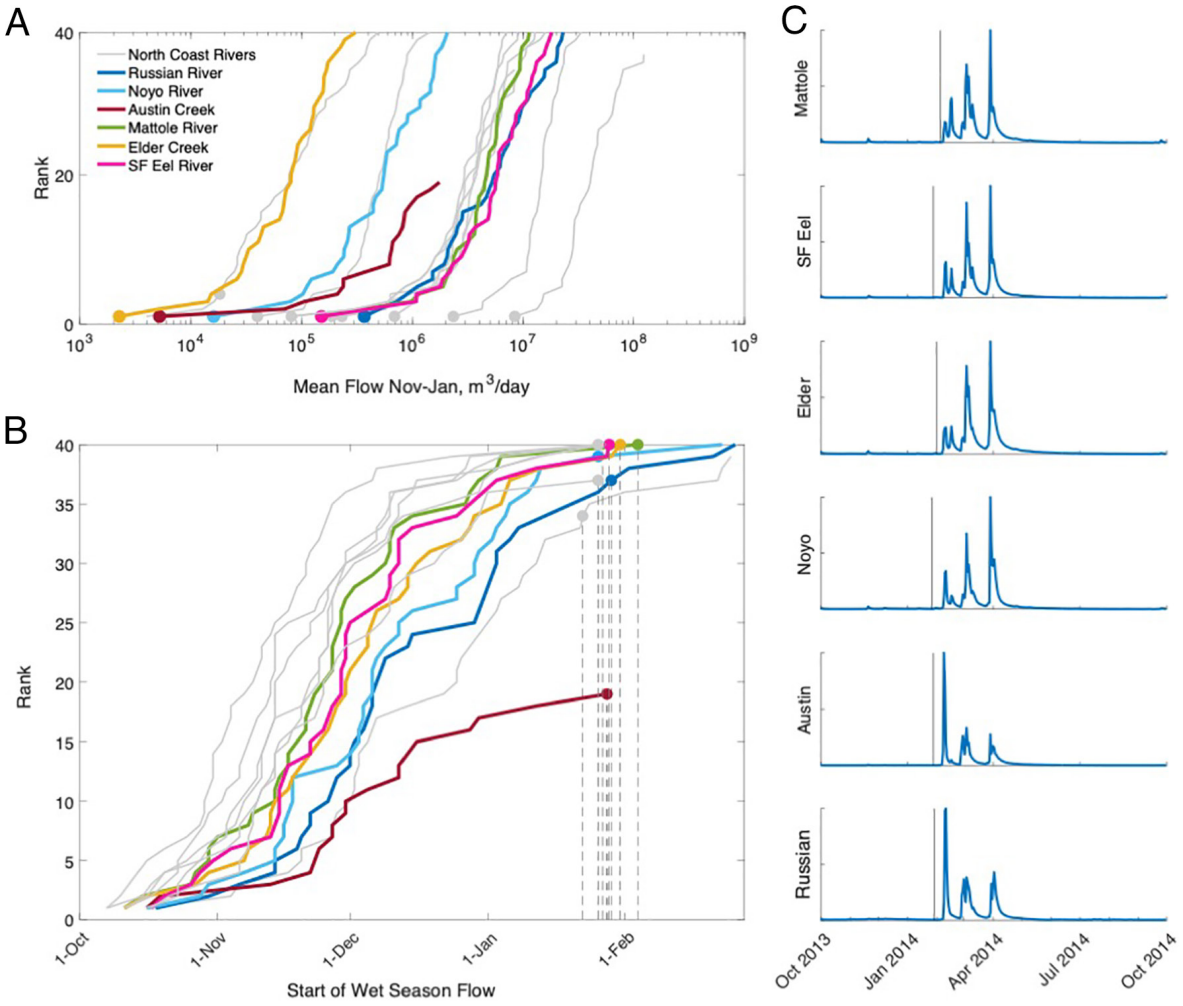
Hydrological conditions during winter 2013 to 14 relative to the historic record. Panel (*A*) shows mean river discharge during November-January for a 40-y period on the horizontal axis (note the logarithmic scale), and the rank order of these flows on the vertical axis. The lower the rank, the more unusually low the flows were. The ranks during winter 2013 to 14 are highlighted with circles. The colored lines and points reflect data from our focal watersheds, whereas gray lines show flow data from seven additional North Coast Rivers for regional context. Panel (*B*) shows the same watersheds and the rank order of the date of winter onset. Here, the higher the rank, the later the onset date. The ranks of the winter onset in 2013 to 14 are highlighted with circles, and the corresponding gray dashed lines indicate the date of the start of the wet season. Panel (*C*) shows 2013 to 14 hydrographs for the focal watersheds organized from north at the *Top* to south at the *Bottom*, where the vertical line indicates the onset of winter flows based on the e-flows calculator (*Materials and Methods*). See *SI Appendix*, Table S1 for information on USGS gages included in our analysis.

During the salmon breeding window over the 40 y analyzed, the lowest mean flows for most watersheds occurred in 2013 to 14, when flows were abnormally low across all study watersheds (Fig. 1*A*). For 12 of 13 gages, the mean flows experienced during the salmon breeding window in 2013 to 14 were the very lowest in the entire long-term record, and were the fourth lowest mean flows for the 13th gage.

Moreover, the onset of 2013 to 14 winter flows was delayed relative to the long-term record in most watersheds (Fig. 1*B*). Winter flow onsets (26) occurred at a similar date across all watersheds, with a mean onset date of January 27, 2014 (range: January 22–February 4, 2014). This onset is approximately 2 mo later than the historical median onset date across the North Coast Rivers, which ranged from 9th November (Smith River) to 26th December (Walker Creek). The winter onset in the subset of systems with biological data ranged from 26 November (Mattole River) to 6 December (Russian River). When analyzing the full range of North Coast rivers, we found that for 9 of the 13 gages, the winter onset in 2013 to 14 was the latest experienced over all 40 y analyzed, and for all watersheds it was within the 7 latest starts to winter.

The widespread and abnormally late winter onset in 2013 to 14 relative to historical conditions (Fig. 1*B*) was also associated with relatively low flows even after flow initiation. In most watersheds, the higher flow conditions that typically mobilize fish did not occur for several weeks after winter rains had commenced, and these higher flow conditions occurred synchronously across the North Coast region (Fig. 1*C*).

### Adults Were Delayed in Their Arrival to Breeding Grounds.

**Fig. 2.**
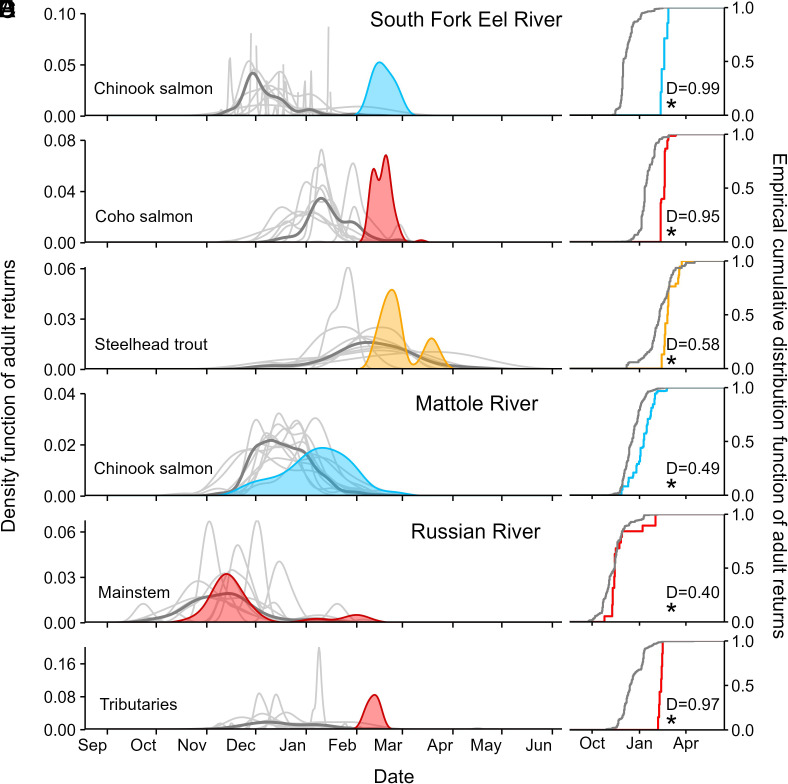
Phenology of adult salmonids. Arrival of adult salmon to the breeding grounds was delayed in winter 2013 to 14 relative to their “typical” timing based on observations from several coastal systems presented as density (*Left*) and cumulative density (*Right*) plots. The vertical axis on the density plots represents the proportion of observations for all arrivals such that the area under each curve totals to 1. Panels (*A*–*C*) represent observations of Chinook, coho, and steelhead from tributaries of the South Fork Eel River, respectively, (*D*) Chinook observations in the Mattole River watershed, and (*E* and *F*) coho salmon in the Russian River watershed. Adult phenologies in the South Fork Eel River were based on observations of salmon nests, whereas observations in the Mattole and Russian River watershed were based on observations of live adults. Moreover, in the Russian River, the observations are based on marked (PIT-tagged) fish. A series of PIT arrays in the Russian River documented a typical arrival timing for PIT-tagged coho salmon to the mainstem (*E*), but delayed access to four intensively monitored tributary breeding habitats due to low flows that restricted access until the early February storms (*F*). Each plot compares the phenology in winter 2013 to 14 with the phenology for a given species and location across “all other years” of monitoring (*SI Appendix*, Table S2). On the cumulative density plots (on the *Right*), we present the Kolmogorov D statistic (*Materials and Methods*). In all cases, the D statistic was significant at α = 0.05, indicating that adult arrivals in 2013 to 14 were significantly delayed relative to the pattern in other years. See *SI Appendix* for details on the monitoring programs (including duration) for each species and watershed combination.

In the South Fork Eel River watershed in Mendocino and Humboldt counties, arrival of adults of all three species to the spawning grounds was delayed relative to the long-term pattern, and synchronized—none of the salmonid species were able to access tributary breeding habitat until a February 10th storm (Fig. 2 *A*–*C*). The result was a contracted breeding period overall, with the normally early arriving Chinook salmon experiencing the largest phenological delay (71 d, Fig. 2*A*), followed by coho salmon (32 d, Fig. 2*B*) and then steelhead trout (17 d, Fig. 2*C*).

In the Mattole River watershed in Humboldt County, arrival and spawning of Chinook salmon was delayed by ~20 d relative to the long-term pattern (Fig. 2*D*). Like many coastal watersheds in California, the Mattole River has an intermittent estuary, meaning that a sand bar blocks fish passage through the mouth of the river during dry periods. The estuary was open (breached) for only two short windows during the fall (30 September-12 October and 19 November-1 December) during which Chinook entered the system. Surveys in late November revealed several hundred fish holding in the lower mainstem (up to river mile 27, see “*Shift in Breeding Locations*” below), but limited evidence of breeding (only three nests were detected by late November, N. Queener, pers.comm.). Little spawning occurred prior to the December 26 to 27 surveys; up until then, only 13 Chinook nests were observed in total, whereas 24 new nests were observed on the December 26 to 27 survey. All spawning upstream of river mile 27 (measured from the mouth of the river) occurred after the rain event on February 8, 2014, which restored access to typical breeding locations upstream.

In the Russian River in Sonoma County, the pattern of adult coho salmon arrival to the mainstem Russian River was slightly delayed in winter 2013 to 14 relative to patterns in other years, based on detections of PIT-tagged fish at a PIT array on the mainstem (Fig. 2*E*). However, the mouths of several tributaries were completely dry until a February 6th storm. Consequently, adult coho were unable to access the tributary breeding grounds until February 6th, based on detections of adults at PIT arrays in the lower reaches of four intensively monitored tributaries (Fig. 2*F*). Only seven adults were detected on these tributary PIT arrays once flow resumed. They were in poor condition (e.g., *SI Appendix*, Fig. S4) and produced very few offspring (“*Reduced and Contracted Patterns of Juvenile Production*”).

These 3 examples all indicated typical breeding locations being used, but with atypical (significantly delayed) timing (Fig. 2).

### Shift in Breeding Locations.

In addition to delayed phenology, the location of salmonid breeding also differed during the winter of 2013 to 14 due to the extremely late rains.

**Fig. 3.**
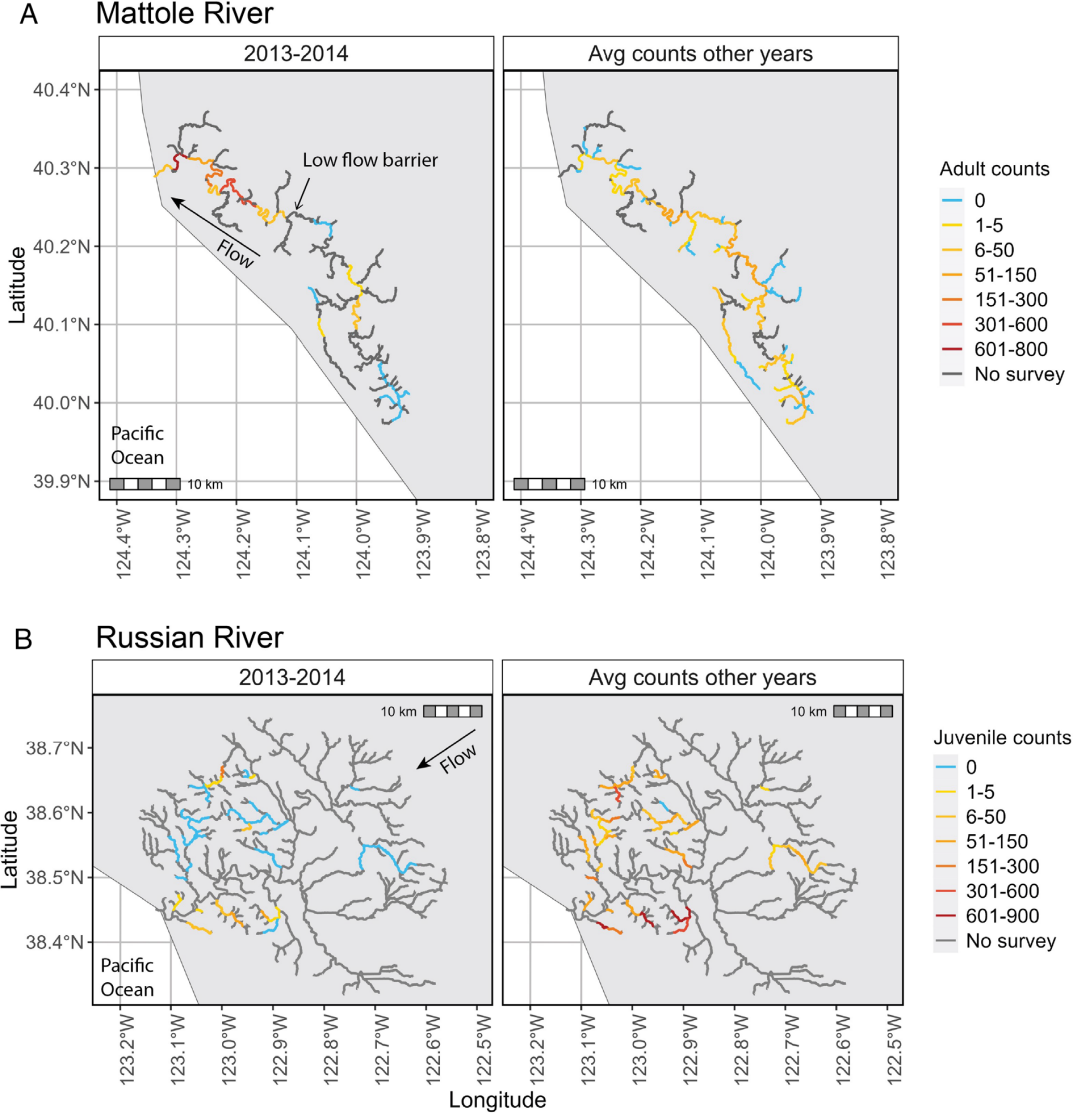
The flow–phenology mismatch in winter 2013 to 14 influenced not only the arrival timing of adult salmonids to the breeding grounds but also the distribution of (*A*) breeding adults and (*B*) juvenile rearing habitat. Panel *A* shows the adult Chinook distribution in the Mattole River system, where two mouth breaches in Fall 2013 allowed adult salmon to access the lower mainstem of the Mattole River but no Chinook made it upstream past a boulder barrier at river mile 27 before flows declined and the river mouth closed again. November, December, and January surveys revealed the highest densities of breeding Chinook salmon downstream of river mile 5 in the lower mainstem, with multiple shallow riffles between the estuary and river mile 24 restricting fish passage. All of the spawning upstream of river mile 27 occurred after the rain event on February 8, 2014, which raised flows sufficiently to allow access upstream of the boulder barrier. In eleven other years of surveying (*SI Appendix*, Table S2), adults were more evenly spread throughout the mainstem Mattole and tributaries. Panel *B* shows that juvenile rearing habitat was greatly contracted in the Russian River Watershed in the summer of 2014 (*Left* panel) as compared to the distribution across other years of study (*SI Appendix*, Table S2). Juveniles were concentrated in only four tributaries in summer 2014, as opposed to 19 to 20 tributaries in the other years of study.

In the Mattole River, the breeding distribution of adult Chinook salmon was shifted down-river in 2013 to 14 compared to the typical pattern. Although some adults entered the river during two mouth breach events in the fall, these fish were confined by low flows to the lower mainstem. Before 8 February, no Chinook were observed spawning upstream of a low-flow boulder barrier at river mile 27. Instead, Chinook were concentrated downstream of river mile 5 from late November until 8 February. Following the February 8 rains, some individuals were observed breeding upstream of the boulder barrier on the mainstem (i.e., some fish eventually spawned in the typical, more upstream location, but with significantly delayed timing; Fig. 3*A*). In surveys during eleven other years, adult Chinook had been more evenly spread throughout the mainstem Mattole and tributaries, including well upstream of the low-flow boulder reach that proved a barrier in 2014 (Fig. 3*A*).

In the South Fork Eel River, California Department of Fish & Wildlife documented widespread mainstem spawning by Chinook salmon during the winter of 2013 to 14, in contrast to most years when most spawning is observed further upstream in the tributaries (S. Ricker, CDFW, pers. comm.). As in the Mattole River, some Chinook eventually accessed the tributaries and spawned in the typical locations further upstream, but with significantly delayed timing (Fig. 2*A*). Chinook in the South Fork Eel mainstem, in contrast, spawned at a more typical time but were atypically concentrated down-river (i.e., in the mainstem).

### Reduced and Contracted Patterns of Juvenile Production.

**Fig. 4.**
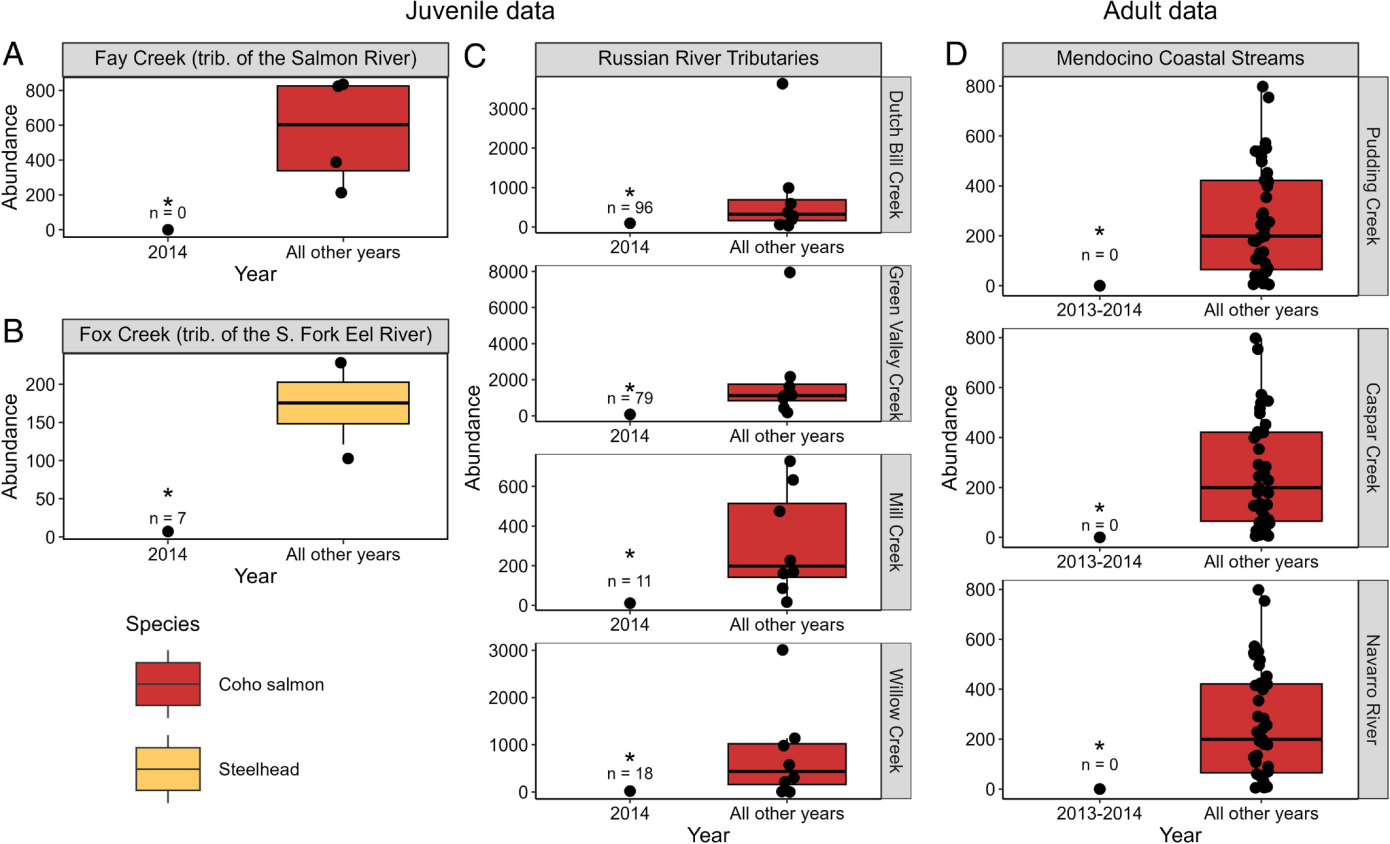
Flow–phenology mismatch during the winter of 2013 to 14 reduced juvenile production across several impacted watersheds and, in three cases, resulted in complete cohort failure. Juvenile production in summer 2014 compared to other years of monitoring (*SI Appendix*, Table S2) in (*A*) Fay Creek (a tributary of the Salmon Creek, Sonoma County, CA), (*B*) Fox Creek (a tributary of the South Fork Eel River, Mendocino County, CA), and (*C*) several tributaries of the Russian River (Sonoma County, CA). In three Mendocino coastal streams, a sandbar blocked the estuary mouth during the 2013 to 14 winter, precluding access by adult coho salmon and leading to cohort failure at the watershed scale (*D*). An asterisk indicates that the abundance estimate for 2013 to 14 fell outside the 95% CI of estimates based on all other years of data collected at that site.

In Fay Creek, a tributary of Salmon Creek in Sonoma County, no age-0 coho salmon were encountered during the 2014 summer survey (Fig. 4*A*), suggesting that adult coho salmon had been unable to access this tributary breeding habitat. In contrast, later-arriving steelhead adults did access Fay Creek, as age-0 steelhead were abundant and distributed throughout the study reach in the summer 2014 survey (*SI Appendix*, Fig. S2). These results contrast with other years when juveniles of both species co-occurred throughout the study reach on Fay Creek (Fig. 4*A*). In contrast, juveniles of both coho and steelhead were widespread and abundant in nearby Tannery Creek in the summer of 2014 (*SI Appendix*, Fig. S2), indicating that not all tributaries in the Salmon Creek Basin were inaccessible to upriver migrating coho salmon during the winter of 2013 to 14.

Juvenile surveys during summer 2014 in Fox Creek (tributary of the South Fork Eel River) suggested that up-river migrating adult steelhead had been unable to access the tributary breeding habitat (Fig. 4*B*). The few juveniles seen were all confined to the uppermost portion of our ~1.3 km study reach, where resident fish (nonanadromous rainbow trout) dominate (27). During the other years of study, juvenile surveys revealed abundant age-0 fish (Fig. 4*B*) that were distributed throughout Fox Creek (27).

In the Russian River (Sonoma County), only five of 25 tributaries surveyed during the summer of 2014 supported juvenile coho, as compared to 12 to 20 tributaries (mean = 15.4 tributaries) in other years of study (Fig. 3*B*). (Because of potential for juvenile dispersal among tributaries, we counted tributaries as supporting juveniles only if > 5 individuals were encountered in surveys.) Moreover, juvenile surveys in four intensively monitored tributaries (Dutch Bill, Green Valley, Mill, and Willow, *SI Appendix*, Fig. S1) revealed extremely low recruitment in 2014 compared to other years of study (Fig. 4*C*).

Together, these results suggest that juvenile rearing habitat was greatly contracted and that recruitment was greatly reduced in 2014 compared to other years of study.

### Cohort Failure at the Watershed Scale.

The California Department of Fish & Wildlife in cooperation with the Pacific Marine Fisheries Commission, Mendocino Redwood Company, and Redwood Timber company have monitored the annual abundance of adult coho salmon in eight coastal Mendocino watersheds for 12 to 15 y (*SI Appendix*, Table S2). Three of these systems have “intermittent” estuaries that open during the wet season and close during the dry season, when sandbars form across their mouths, severing connection to the ocean until the fall rains. During the winter of 2013 to 14, the sandbars remained fully intact in Pudding, Caspar, and Navarro watersheds. Following the early February storms, surveyors encountered no adult females nor nests in the Pudding, Caspar, and Navarro watersheds (Fig. 4*D*). Juvenile sampling during the following summer confirmed cohort failure (i.e., zero recruitment) in these three watersheds.

### Long-Term Implications of Population Loss.

To explore the long-term implications of population loss due to the 2013 to 14 flow–phenology mismatch, we tracked progeny of this cohort through time and also evaluated the age composition of a future cohort potentially affected by the event. We focused on coho salmon from the Russian River—a population complex supported by a captive broodstock program and a large-scale monitoring program that tracks the fates of individually marked fish in four intensively monitored streams (28). First, we used data from this system to follow the fate of offspring from the 2013 to 14 breeding population. At their southern range boundary, coho salmon have a relatively simple life history, with most individuals spending 1.5 y in freshwater and 1.5 y in the ocean before breeding at age 3. However, some individuals breed at age 2 after only 6 mo at sea. Breeding at age 4 is exceedingly rare in the region. Thus, coho born in the spring of 2014 potentially returned to breed across two adult return years: winter of 2015 to 16 (at age 2) or 2016 to 17 (at age 3). We found no evidence that individuals born in 2014 returned to breed at age 2 (in 2015 to 16) and estimate only six individuals born in 2014 survived to breed at age 3 (*SI Appendix*, Fig. S8*A*). In short, the 2013 to 14 cohort was effectively lost, reducing the number of potential breeders during the winters of 2015 to 16 and 2016 to 17.

To evaluate whether an adjacent in time cohort rescued the 2016 to 17 breeding population (when coho born in 2014 were expected to return as age-3 adults), we explored the contributions of age-2 adults (offspring of the 2014 to 15 cohort). We found very limited contributions of age-3 fish (see above) or age-2 adults (estimated: n = 8 fish) in the 2016 to 17 breeding population. In short, the 2016 to 17 cohort was also effectively lost (*SI Appendix*, Fig. S8*B*). This result underscores the influence of the multiyear drought on brood years adjacent in time in reducing the potential for buffering through age complexity and overlapping generations. While this population was not rescued by age complexity, it was rescued by reserves from a captive broodstock program, which exists precisely because the Russian River population complex is vulnerable to extinction (28).

## Discussion

During California’s multiyear 2012 to 16 drought, the winter of 2013 to 14 stands apart because the first large storms during the wet season arrived extremely late (in February 2014 as opposed to October or November in more typical years, Fig. 1). The delayed rains affected three species of up-river migrating salmonid fish—but the impacts differed across species due to differences in their breeding phenology (Fig. 2 and *SI Appendix*, Table S3). Adult Chinook, coho, and steelhead were unable to access some tributary breeding sites due to low flows, leading to contracted breeding and rearing distributions (Figs. 3 *A* and *B*, respectively); reduced juvenile production (Fig. 4 *A*–*C*); and, in three extreme cases, complete cohort failure at the watershed scale (Fig. 4*D*). Documenting these place-based, event-driven changes explains why different rainfall patterns have different impacts on riverine species with different phenologies. These observations illustrate how extreme events can push stressed species, like California coho, over the brink of extinction locally, contributing to the erosion of range boundaries.

During wet years, dozens of atmospheric river events reach California (29), whereas in dry years, only a handful of significant storms occur. When there are only a few large storms, the timing of particular storms will play an outsized role in determining which upriver migrating species are able to access their typical breeding grounds at the typical time. While we focus here on consequences of flow–phenology mismatches for breeding adults, these mismatches can also strongly influence the survival of emerging fry (30), and likely constrain salmon population dynamics throughout their life cycle. With changing rainfall patterns in California, including more variable rainfall timing (31), flow–phenology mismatches are likely to become more common, with potentially different outcomes across species in years with different rainfall patterns. Indeed, life histories of many riverine organisms are synchronized with the long-term river flow patterns (16), suggesting that flow–phenology mismatches could disrupt life history strategies throughout river food webs with widespread consequences for river ecosystems. Natural selection due to flow–phenology mismatches could drive adaptive changes in breeding timing that mitigate the impacts of delayed rains in future years. However, we observed no shift toward later arrival timing in the offspring of the adults impacted by delayed rains in 2013 to 14 (*SI Appendix*, Fig. S7). Notably, the onset of winter rain has become progressively later in California (32), suggesting potential for progressively later rains to drive selection favoring later adult return timing, much as consistently reduced flows could select for smaller adult salmon body size (33).

The relative success of migratory fishes under climate change will be determined not only by differences in phenology relative to rainfall timing but also by where species breed within river networks. Chinook salmon often breed in low-gradient river mainstems, whereas coho salmon and steelhead typically breed in steeper smaller tributaries upstream. Access to these tributary streams by breeding adults can be impeded during low flow periods by barriers such as alluvial fans deposited at confluences with mainstems or by “hanging tributaries” (knickpoints or small waterfalls that are impassable at low flow) that form where tributary incision rates have not kept up with downcutting in mainstems (34, 35), as is commonly the case along the tectonically active west coast of North America. In contrast, mainstems are more likely to remain accessible despite flow variation, except in the case of bar-built estuaries when the entire watershed is cut off from the ocean. In the Mattole River, Chinook spawned at very high densities in a short reach of the lowermost mainstem that was accessible during the dry winter of 2013 to 14 (Fig. 3*A*). Salmonids that breed in tributary streams such as coho salmon and steelhead trout may be more likely to lose access to breeding habitats when low flows occur during the breeding season; delayed onsets of winter are most likely to impact coho salmon, whereas dry springs are more likely to impact steelhead trout due to their later phenology.

Mismatches between weather conditions and phenology that influence population dynamics (36) can also accelerate range expansions. Warmer winters with reduced winter mortality of mountain pine beetles have enabled their northward expansion (37), and warm conditions at the right places and times facilitate excursions of Pacific salmon into the Canadian Arctic (38). In contrast, our work suggests that increasingly severe interannual variation in precipitation patterns will create windows of unsuitable conditions at the trailing range edges, potentially contributing to the erosion of those range margins.

Climate change is triggering more frequent extreme events, which can have sudden and sometimes persistent effects on ecosystems. While extreme events are by their nature hard to study, long-term studies and distributed monitoring efforts provide opportunities to document their impacts. A 30 y study of desert rodents revealed that extreme floods cause catastrophic mortality, altered species interactions, and ultimately restructured the community (39). A long-term study on *Anolis* lizards revealed that hurricanes cause strong episodes of natural selection on lizard morphology that persist across generations (40). Extreme events can also drive sudden changes in species’ distributions. For example, a heatwave caused widespread mortality of a habitat-forming seaweed, contracting its global distribution by 5% at its warm edge (11). How enduring a range change must be before it is accepted as a range change rather than a temporary population extirpation is not entirely clear, but these authors argue that the nearest population source is below major ocean currents, making recovery unlikely [i.e., a true shift, (11)]. In the case of drought-affected California salmonids, the loss of access to breeding habitat was confined to a single year, and populations were rescued by age complexity and reserves in the ocean or, in one extreme case, a conservation broodstock program (Russian River). However, such sudden and large reductions in population productivity can leave trailing-edge populations vulnerable to extinction due to demographic stochasticity. Moreover, sequential disturbances (such as consecutive years of flow–phenology mismatch or other disturbances) that impact adjacent in time cohorts hold considerable potential for causing long-term population losses and range erosion of imperiled species. For example, most endangered coho salmon in California breed at age 3, suggesting three consecutive years of recruitment failure is sufficient to extirpate coho from a given breeding location. In our case, the delayed rains in 2013 to 14 were part of a longer, multiyear drought that impacted California between 2012 to 16. Conditions supporting salmon in freshwater were generally poor during the multiyear drought (41) and the drought coincided with the 2014 to 16 marine heat wave (42), which likely reduced the success of salmon at sea. Taken together, these patterns highlight the importance of placing extreme events in a multiyear context to understand their long-term impact on species with complex life histories.

The extremely late rains and flow–phenology mismatch during the 2013 to 14 salmon breeding season provide a glimpse into the importance of extreme events in causing sudden population loss and stepwise distributional changes in imperiled populations near their range edge—in this case, a contraction down-river within watersheds (e.g., Fig. 3*A*) or loss of access to entire watersheds (Fig. 4*D*). We observed multiple scales of range contraction within river ecosystems, ranging from loss of subpopulations within watersheds (Fig. 4 *A*–*C*) to cohort failure at the watershed-scale (Fig. 4*D*). We posit that losses within and among watersheds of subpopulations with different phenologies or other traits sensitive to climate variation (7) will erode range boundaries, underscoring the importance of considering internal (subpopulation) structures of geographic ranges in studies of range redistribution (43). While prior work has emphasized that population declines precede range shifts (e.g., ref. 44), our work suggests that loss of spatially structured complexes (e.g., metapopulations) at species edges may be hastened by extreme events that cause simultaneous collapse of multiple populations and reduce rescue potential. Salmon resilience to climate variability and extremes is further reduced by flow–phenology mismatch events that concentrate adults, and hence juveniles, into a subset of sites accessible during the breeding season. This spatial contraction potentially erodes the natural buffering capacity that emerges when salmon populations are more widely distributed and exposed to partially decoupled environmental conditions (e.g., refs. 45 and 46).

The flow–phenology mismatch documented here occurred within the context of a multiyear drought. Drought conditions, and multiyear droughts in particular, can impact the spatial patterning of adult salmon in river systems (and, hence, juvenile distributions), as well as the stage-specific survival of those juveniles (47). However, the impacts of drought will be mediated by the local environment [and, in particular, how the landscape filters climate, (48)], suggesting that drought conditions—to a point—are likely to generate heterogeneous responses across salmon populations, even if overall survival is reduced (41, 47). In contrast, flow–phenology mismatches are more likely to cause simultaneous reductions in multiple populations, which reduces potential for demographic rescue from nearby populations. When such sudden losses occur in population complexes that are already reduced and simplified at range boundaries, likelihood of rescue via dispersal or life history diversity is further reduced, making permanent range contraction more likely.

Salmonids in California impacted by the 2013 to 2014 extreme event all returned to the impacted sites in subsequent years (*SI Appendix*, Figs. S5 and S6), rescued by reserves in the ocean, life history diversity, and, in one case, a conservation broodstock program. While the three salmon returned to impacted sites in years with better flow conditions, future drought and periodic loss of habitats where spawning adults require several high flow events for access, may erode population resilience. This may be particularly true along the trailing edge of a species’ biogeographic range, where altered hydrologic timing and extreme events can drive mismatches, such as those reported here, between phenology and environmental conditions, triggering sudden population losses. Trailing edge populations warrant special vigilance and protection, because they may harbor genetic or epigenetic variation (e.g., ref. 49) useful for adaptation to a warming world.

## Materials and Methods

### Study Sites and Species.

Coho salmon (*Oncorhynchus kisutch*), Chinook salmon (*O. tshawytscha*), and rainbow and steelhead trout (*O. mykiss*) are three species of salmonid fishes distributed around the Pacific Rim (22). California represents the southern end of the latitudinal range for both salmon species and for the ocean-migrating form of *O. mykiss* (“steelhead”). Population complexes of all three species in California are listed for protection under federal and state Endangered Species Acts [summarized in (50)]. All three species breed in rivers, and their offspring migrate to the ocean after spending a variable length of time in fresh water, ranging from months to years. After feeding and growing in the ocean, adults migrate back into freshwater to breed. The three species differ in their breeding phenology, with Chinook salmon typically breeding earliest (~November-December), followed by coho salmon (~December-January), and with steelhead breeding relatively later, and with the most protracted breeding window (~December/Jan-March/April), see Fig. 2. Breeding location often differs among species due to differences in habitat preferences and temporal shifts in locations of given sets of preferred conditions [gravel size, water depth, velocity, and temperature (51)]. In general, larger species like Chinook, which tend to occur in lower elevation (higher-order) rivers, can build nests in relatively faster and deeper waters (and dig deeper nests that are less prone to scour), whereas smaller species like coho tend to build nests in smaller tributaries in reaches with slower and shallower waters. Moreover, breeding phenology affects a spawner’s access to certain parts of the watershed. Because Chinook breed the earliest, at the start of the wet season, they are often confined to mainstems or larger tributaries that are accessible even under low flows. Coho breed slightly later in the wet season and are relatively smaller, so can access breeding sites in smaller channels that are further upstream. Steelhead breed the latest, are better jumpers, and can often access the uppermost and highest-gradient tributaries late in the wet season (52). We expected differences in the vulnerability to flow–phenology mismatches to emerge as a consequence of breeding phenology and breeding locations.

### Hydrological Data.

Under California’s Mediterranean seasonality, most rainfall occurs between October and April, but the timing and magnitude of rainfall within the wet season varies among years. Most precipitation is delivered in a few large storm events interspersed with smaller storms. Drought conditions emerge when a ridge of high atmospheric pressure off the coast diverts storms north of California (29).

Daily flow data were retrieved from USGS for 13 watersheds in northern California, including coastal watersheds from Marin County to Del Norte County (Fig. 1 and *SI Appendix*, Table S1). The magnitude and timing of flows from the 2013 to 14 winter were compared to the long-term record (specifically, the most recent 40 complete water years 1983 to 2022, except in two cases: Walker Creek, where gage records commenced after 1983 leading to inclusion of 39 complete water years, and Austin Creek, where the gage was reactivated in 2004, leading to inclusion of 18 complete water years). A water year in California is defined as October 1–September 30 (e.g., the 1983 water year corresponds to the period October 1, 1982, to September 30, 1983). We include gage data from Russian River, Austin Creek, Noyo River, Elder Creek, South Fork Eel River, and the Mattole River as representative of conditions in systems where we also have biological observations. Additionally, we present hydrologic data for 7 additional watersheds that spanned a north–south gradient of coastal northern California watersheds, providing a broader regional context for the conditions experienced during the 2013 to 14 winter. See *SI Appendix*, Table S1 for a summary of gage data included in our analysis.

We considered the typical adult salmonid migration window to extend from 1 November to 31 January, capturing the onset of migrations for the three salmonids (Chinook, coho, and steelhead trout) in the study region. For each of the 13 watersheds, we computed the mean flow for this window for each winter across the 1983 to 2022 water years, in each watershed, and then rank-ordered the observations. The 2013 to 14 conditions were identified within the ranked conditions. Here, the lower the rank, the more unusually low the flow conditions were.

For each of the 13 watersheds, we used the e-flows Functional Flows Calculator to identify the onset of winter flow conditions within a given water year using the *ffcAPIClient* package in R (version 0.9.8.3, https://github.com/ceff-tech/ffc_api_client). This tool applies a smoothing function to daily flow data to detect the timing of the transition between the dry season and wet season, when flows begin to increase to their annual maxima (26). We obtained the winter onset timing for each year from 1983 to 2022. The dates were then ranked from smallest to largest using data from the 1983 to 2022 water years, again identifying the 2013 to 14 conditions within these ranked conditions. Here, the higher the rank, the later the winter onset date.

### Biological Data.

We used a bootstrapped Kolmogorov–Smirnov test to determine whether adult arrival timing in 2013 to 14 was delayed relative to the other years of study (where the cumulative distribution for all other years was calculated from the pooled dataset across years). We used ks.boot() function in the R package “Matching” (53) with 1,000 bootstraps. The test returns the Kolmogorov D (“distance”) statistic and associated *P*-value (in our case, for a one-tailed test). We present the D statistics in Fig. 2, which represents the degree of difference measured as the maximum vertical distance between the reference distribution (all other years) and the 2013 to 14 distribution.

To determine whether the juvenile or adult abundance was significantly reduced in 2013 to 14 relative to other years of study, we asked whether the abundance estimate fell outside the 95% CI of estimates based on all other years of data. The 95% CI was estimated by the mean ± SE. SE was calculated as the SD divided by square root of the sample size.

All analyses of biological data were performed in R (54).

## Supplementary Material

Appendix 01 (PDF)

## Data Availability

Summary of datasets and workflows have been deposited in Dryad (https://doi.org/10.5061/dryad.6wwpzgn72) (55). All other summaries are included in the *SI Appendix*. Previously published data were used for this work (56).
