# Supplementary material for: Anatomy of a range contraction: Flow–phenology mismatches threaten salmonid fishes near their trailing edge
Source: Proc Natl Acad Sci U S A. 2025 Mar 31;122(14):e2415670122. doi: 10.1073/pnas.2415670122 (PMC12002297; doi:10.1073/pnas.2415670122)
Supplement: Supplementary file 1 — Appendix 01 (PDF) [file pnas.2415670122.sapp.pdf]

PNAS

Supporting Information for
Anatomy of a range contraction: Flow-phenology mismatches threaten salmonid fishes near their trailing edge

Carlson, S.M., Pregler, K.C., Obedzinski, M., Gallagher, S.P., Rhoades, S.J., Woelfle-Hazard, C., Queener, N., Thompson, S.E., and Power, M.E.

Corresponding authors: Mary Power, Stephanie Carlson
Email: mepower@berkeley.edu, smcarlson@berkeley.edu

This PDF file includes:

Supporting text
Figures S1 to S8
Tables S1 to S3
SI References

Supporting Information Text

Study systems and methods

Fox Creek is a tributary of the South Fork Eel River located on the UC Angelo Coast Range Reserve in Mendocino County and is considered a perennial breeding stream for steelhead trout (*Oncorhynchus mykiss*). Fox Creek has been the site of numerous studies on the ecology of juvenile steelhead (e.g., 1 [data collected in 2010], 2 [data collected in 2012]). SJR surveyed the distribution and abundance of juvenile *O. mykiss* across four summers, 2014-2017, and those data are presented in our analysis.

Fay and Tannery creeks are tributaries of Salmon Creek in Sonoma County. Coho salmon were extirpated from Salmon Creek in the 1990s, but a reintroduction effort is underway as part of the larger Russian River Coho Salmon Captive Broodstock Program (3). Each winter since 2008, adult coho salmon have been released into the Salmon River Estuary to migrate upstream volitionally and select breeding habitat. Fay and Tannery Creeks support adult breeding and juvenile rearing. Juvenile surveys for coho and steelhead have been conducted sporadically following the start of the reintroduction program, and effort increased from 2009-2013 when the Gold Ridge Resource Conservation District conducted juvenile salmonid abundance and distribution surveys during summer. From 2012-2016, CW-H surveyed the distribution and abundance of juvenile *O. mykiss* and *O. kisutch* during summer surveys on Fay and Tannery creeks, and those data are presented in our analysis.

The South Fork Eel River in Mendocino and Humboldt counties is a major tributary of the Eel River in northern California. Tributaries and the upper mainstem of the South Fork Eel River support breeding populations of coho, steelhead, and Chinook. Personnel from the California Department of Fish & Wildlife survey ~37 reaches of this spawning habitat each winter since 2010 following the California Coastal Salmonid Monitoring Program (CMP) protocol (Adams et al. 2011). Under this scheme, biologists conduct spawning ground surveys during the breeding season within a fixed sample frame under spatially balanced probabilistic rotating panel design to estimate annual redd abundance. The results of the surveys are included in annual monitoring reports available by request. The sample frame and survey protocol for the South Fork Eel River were primarily designed to estimate coho salmon redd abundance and although there is substantial spatial and temporal overlap with other adult breeding populations of salmonids, redd abundance estimates for other species are only partially representative of their respective populations. As part of a pilot program in the winter of 2013-14, the survey area was expanded to include portions of the mainstem South Fork Eel River (S. Ricker, pers. comm.).

The Russian River in Sonoma County is home to a large conservation effort targeted at restoring endangered coho salmon (3–5). The monitoring program involves PIT-tagging juvenile coho in multiple tributaries, and detecting their down-river migration as smolts and up-river migration as adults via a series of stationary antenna arrays (hereafter "PIT array"). A PIT array deployed at Duncan Mills on the mainstem Russian River detects movements on the mainstem, including when adults first move upstream from the ocean to the river. PIT arrays located at tributary mouths detect when adults move from the mainstem to their tributary breeding habitat. Detections at the suite of PIT arrays have revealed that adult coho salmon typically move from the ocean into the mainstem Russian River beginning in October (Fig. 2E), and then move into the tributaries in mid-November following the first large storms of the wet season (Fig. 2F). Beginning in 2014, the monitoring program expanded to include summer snorkel surveys in 35-45 tributaries throughout the watershed to estimate coho occupancy patterns and recruitment success (see Figs. 3B and 4C).

The California Department of Fish & Wildlife in cooperation with the Pacific Marine Fisheries Commission, Mendocino Redwood Company, and Redwood Timber company have monitored steelhead, coho salmon, and Chinook salmon in 23 watersheds in the Mendocino Coast sample frame since 2008 following the California Coastal Salmonid Monitoring Program (CMP) protocols

(6). Under this scheme, biologists conduct regionwide spawning ground surveys during the breeding season within a fixed sample frame using spatially balanced probabilistic rotating panel design to estimate annual redd and adult abundance. Life cycle monitoring occurs in several of these systems to estimate smolt abundance, and evaluate growth and survival at various life stages. We included a subset of these data focused on coho salmon from nine watersheds, including Ten Mile River, Pudding Creek, Noyo River, Caspar Creek, Big River, Little River, South Fork Albion River, Navarro River, and Garcia River. Adult escapement estimates for three systems that experienced complete cohort failure are presented in Figure 4D (Pudding Creek, Caspar Creek, and the Navarro River), a determination based on a complete absence of breeding adults, nests, and juveniles. Estimates for the remaining systems are presented in Fig. S3.

The Mattole Salmon Group has conducted annual spawning ground surveys in the Mattole River watershed since 1981, with support from the Bureau of Land Management, CDFW, and private foundations. Since the winter of 2011, the survey effort has used the CMP protocols (6). Wading or boating surveys to enumerate live fish, carcasses, and redds are conducted every 7-14 days, streamflow and visibility permitting, throughout the Chinook salmon spawning season in 15-25 stream reaches selected from a sample frame that includes all the physically accessible potential Chinook salmon spawning habitat in the watershed. Adult phenology and breeding locations are presented in Figs. 2D and 3A, respectively.

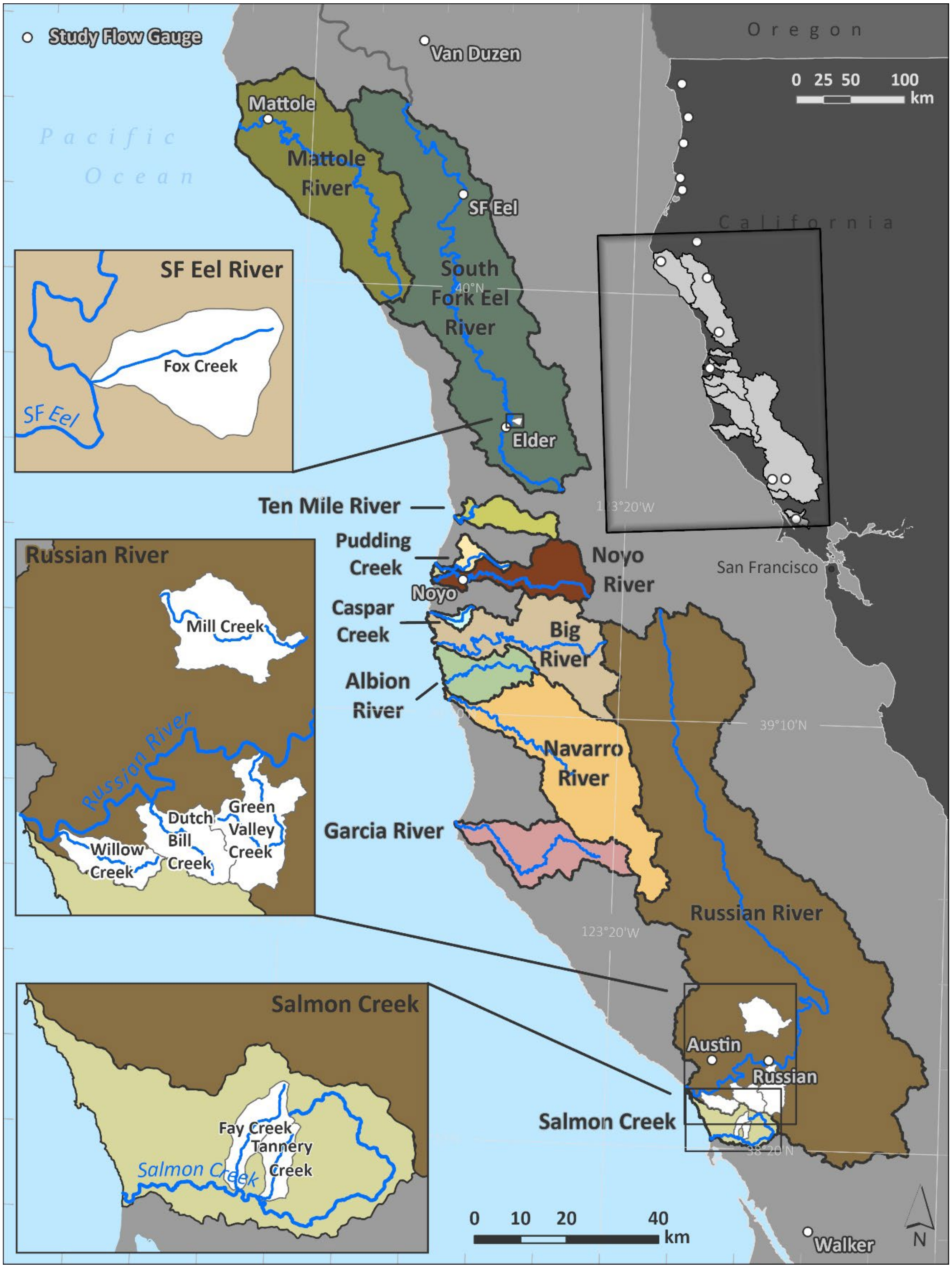

Fig. S1. Focal watersheds in northern California, increasing in scale from left to right. Center colored maps show the watersheds where we have biological observations (insets on the left indicate the specific sub-basins where biological observations were collected) (Table S2). On the right, we locate our focal watersheds (in light grey) along the California coast; white dots indicate the locations of USGS gages (Table S1) from which we downloaded flow data for analyses.

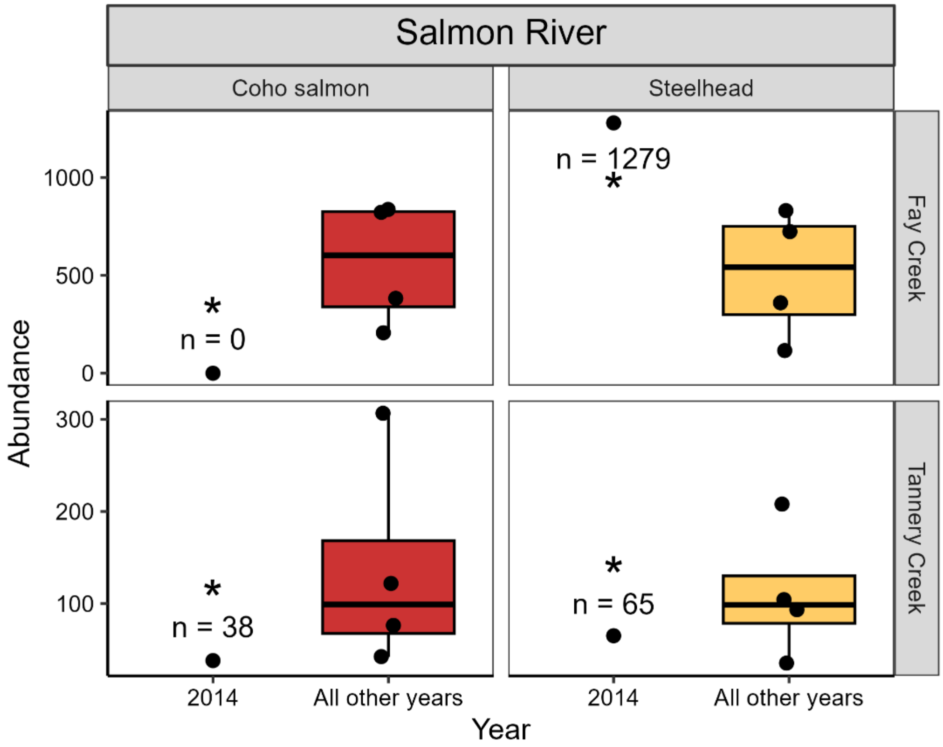

Fig. S2. Within the Salmon River (Sonoma County, CA), both coho and steelhead typically breed in tributaries including Fay and Tannery Creeks. During the winter of 2013-14, early arriving adult coho salmon were apparently unable to access Fay Creek, while later arriving steelhead were able to access Fay Creek. In contrast, both species were able to access Tannery Creek in the winter of 2013-14, perhaps suggesting differences in mouth morphology contributed to spatial variation in access to breeding habitat. An asterisk indicates that the abundance estimate for 2013-14 fell outside the 95% CI of estimates based on “all other years” of data collected at that site.

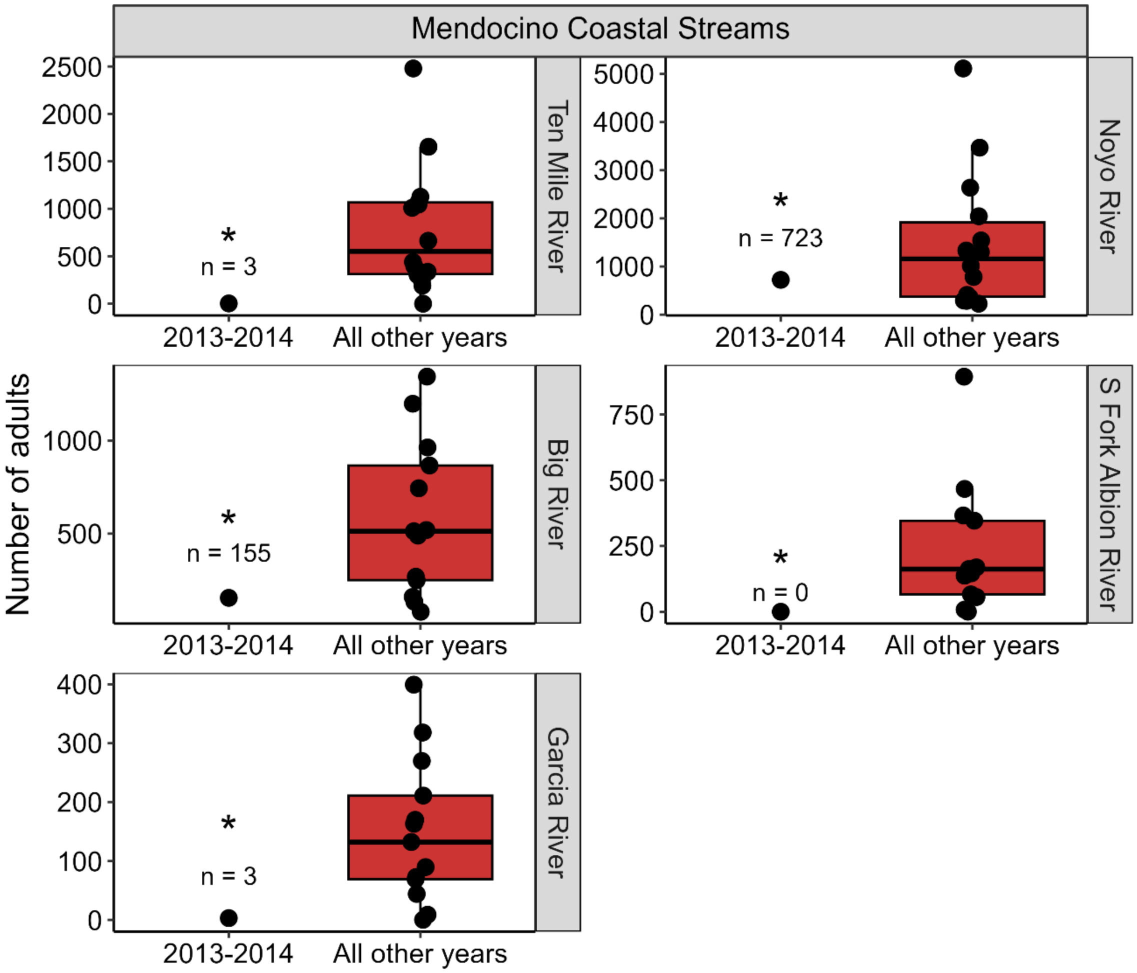

Fig. S3. Flow-phenology mismatch during the winter of 2013-14 resulted in complete cohort failure at the watershed scale in three Mendocino coastal systems due to a sandbar at the estuary that blocked access to the watershed (Pudding, Caspar, and Navarro, see figure 4). While the other monitored Mendocino coastal systems have open estuaries, adult production was also reduced at these sites, presumably as a consequence of upriver low flow barriers that limited adult passage. An asterisk indicates that the abundance estimate for 2013-14 fell outside the 95% CI of estimates based on “all other years” of data collected at that site.

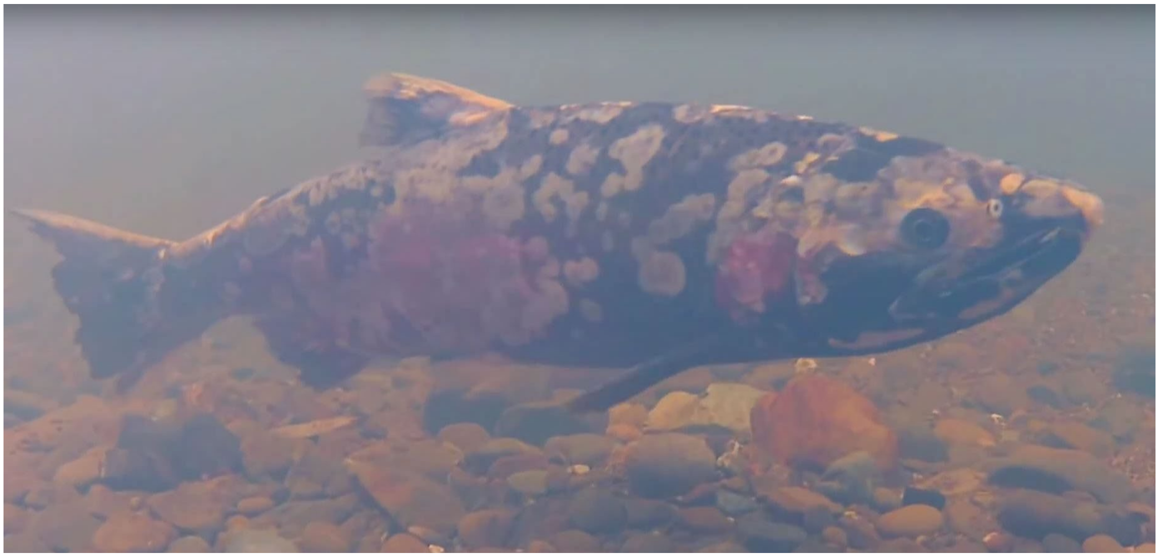

Fig. S4. Photograph of an adult coho salmon holding near the mouth of a tributary breeding site in the Russian River watershed during the winter of 2013-14 when winter rains were delayed. Adults unable to access tributary breeding habitats due to flow disconnection delayed breeding by multiple weeks and were in extremely poor condition when they reached the tributaries following rains in early February that restored tributary access and mobilized the fish. Photo credit: California Sea Grant.

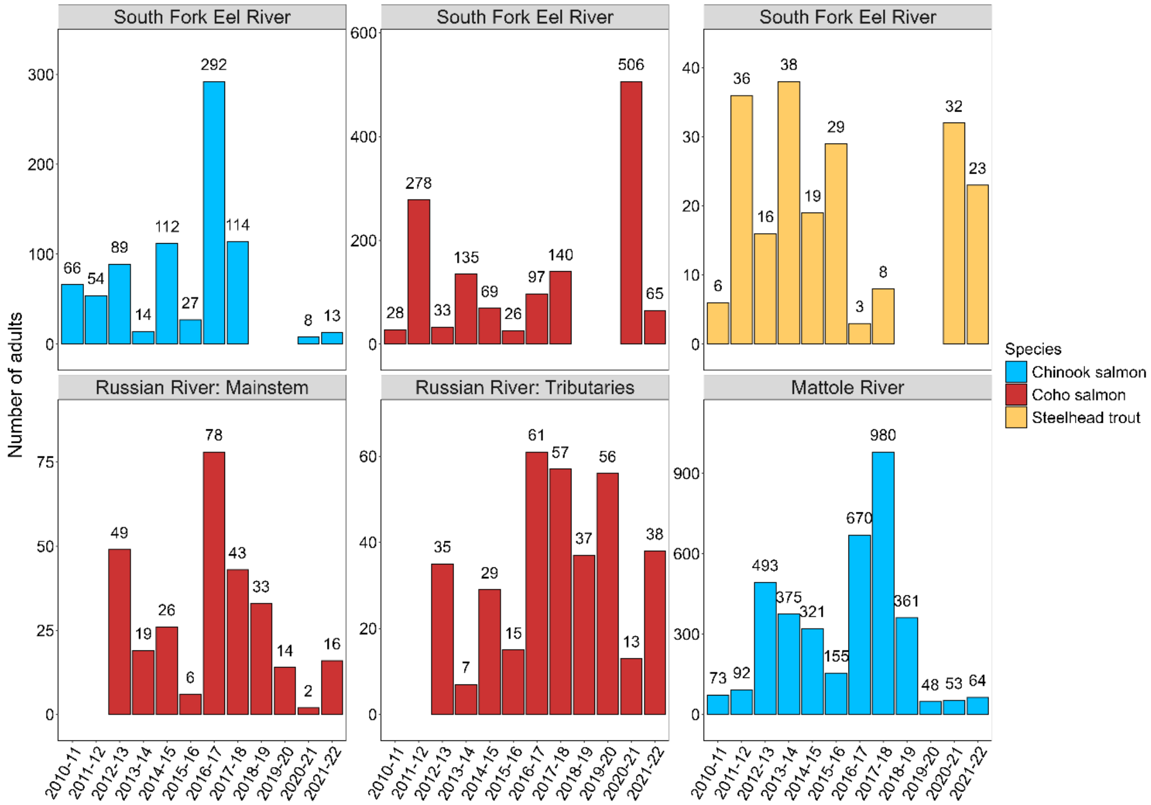

Fig. S5. Adult abundance for the South Fork Eel River (top row), Russian River (mainstem and tributaries), and Mattole River. Note that for the Russian River, he number of adults represents the count of PIT-tagged individuals detected on PIT arrays.

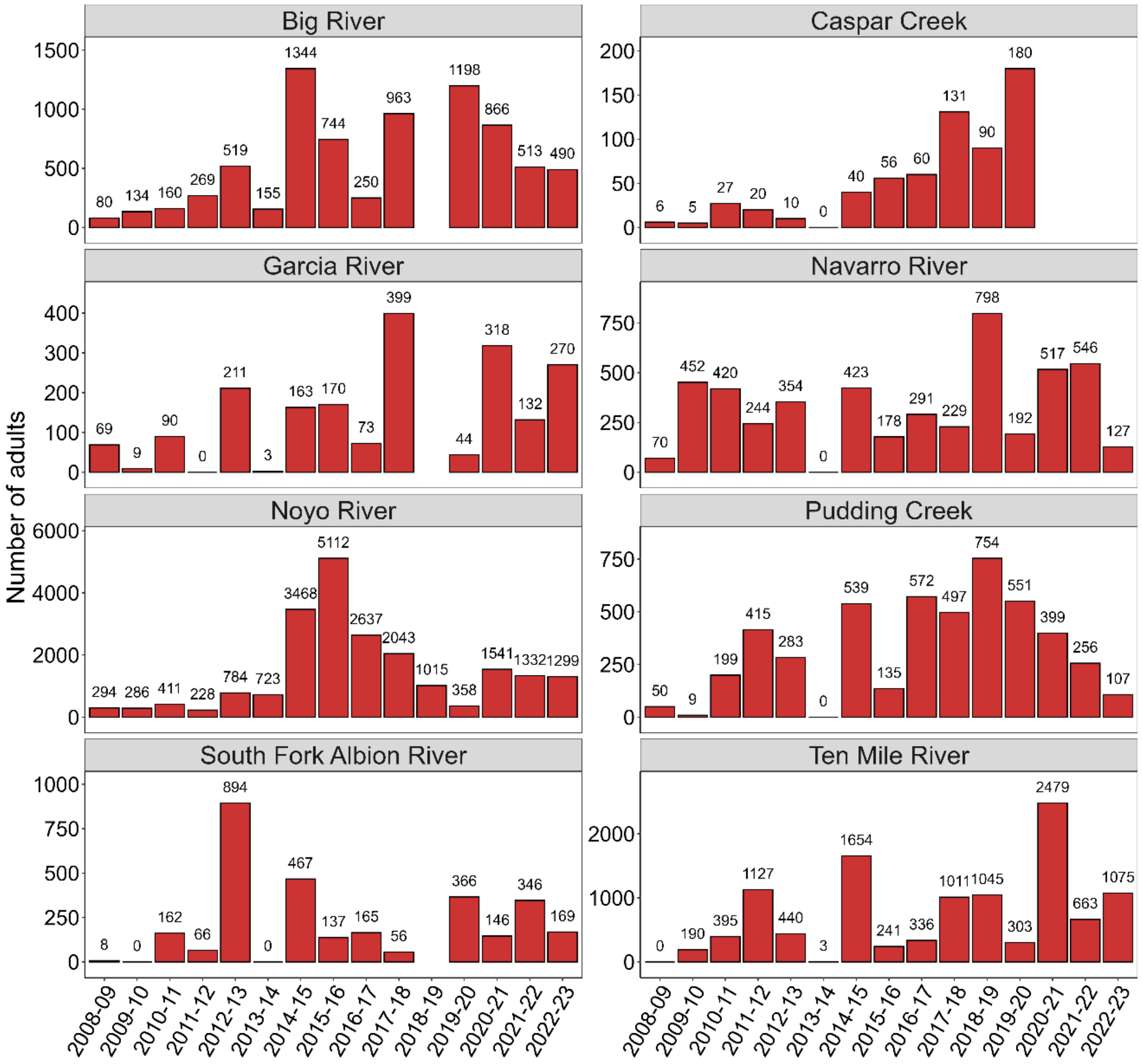

Fig. S6. Time series of adult abundance by year for the suite of Mendocino coastal streams. All of these data represent counts of coho salmon.

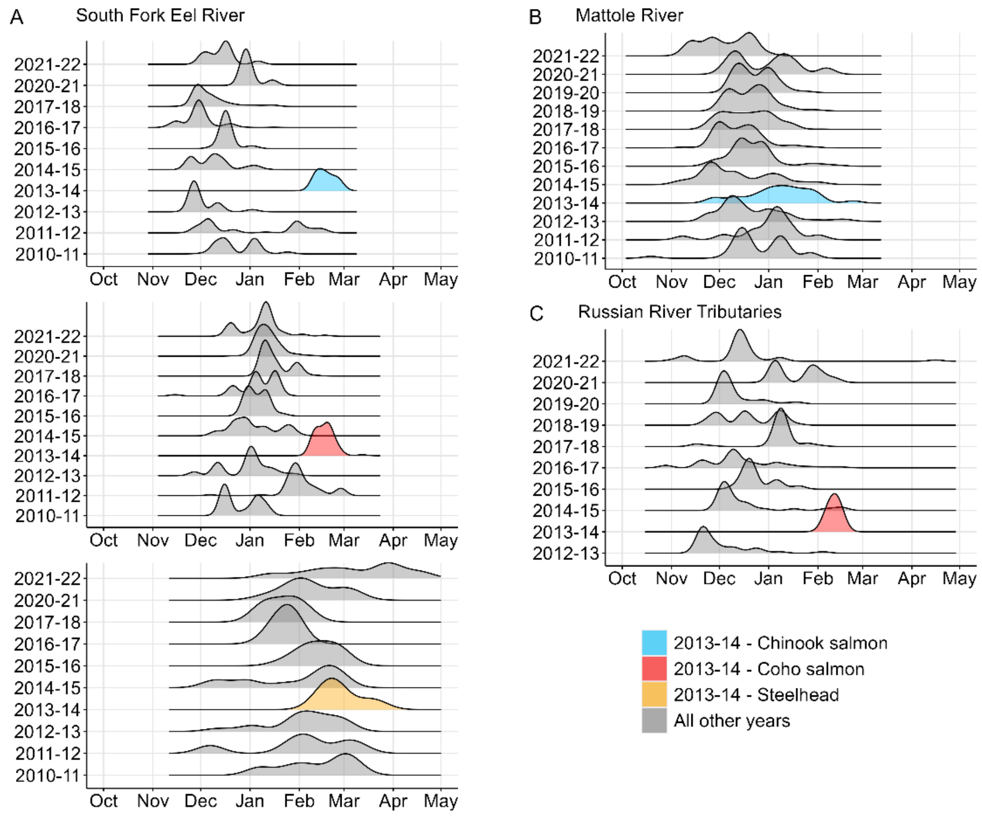

Fig. S7. Adult return timing by year for salmon returning to the South Fork of the Eel River (A, left column), Mattole River (B), and Russian River (C). Results presented separately by species (see legend).

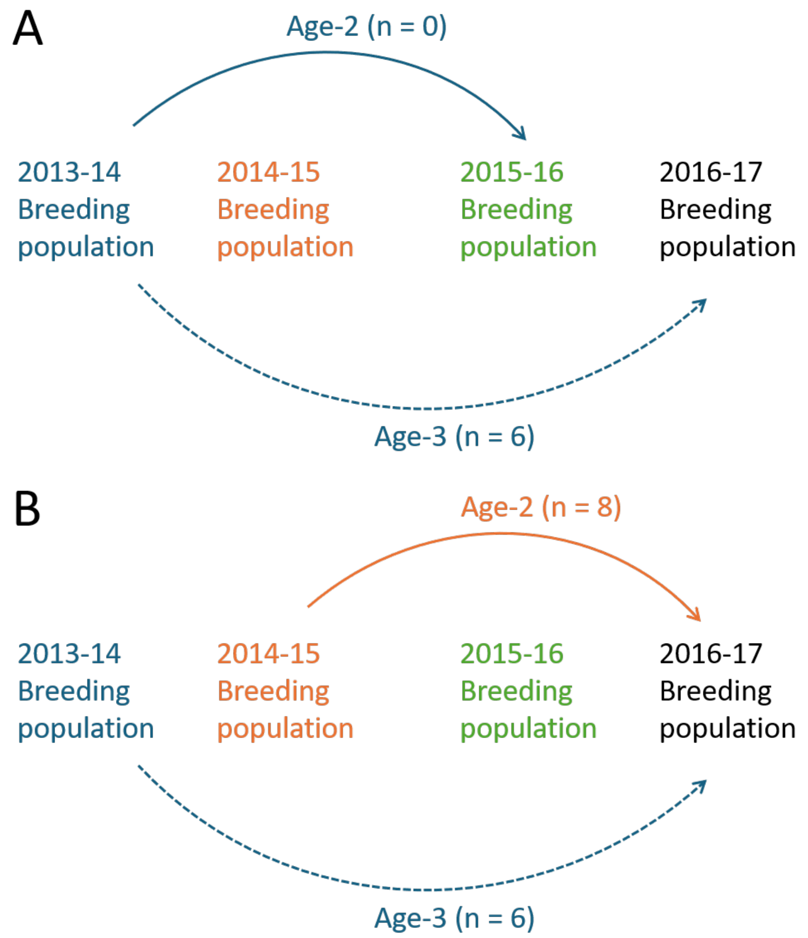

Fig. S8. Example from Russian River showing the long-term impact of the 2013-14 flow-phenology mismatch. In particular, (A) the 2013-14 cohort was effectively loss – no juveniles born in 2014-15 survived to breed at age-2 (in 2015-16) and only 6 individuals survived to breed at age-3 (in 2016-17). We expected the long-term impact of the 2013-14 flow-phenology mismatch to manifest three years later in 2016-17 because most coho salmon breed at age-3. However, fish born in 2014-15 could potentially rescue the 2016-17 breeding population. Fish born in 2015 experienced drought conditions, but were not directly impacted by the 2013-14 flow-phenology mismatch. However, we found very limited contributions of age-2 (born in 2015) or age-3 (born in 2014) to the adult breeding population, highlighting that the breeding population was not rescued by age complexity. Fortunately, another source of reserves existed in the Russian River case – reserves from a conservation broodstock hatchery, which served as the source of the vast majority of adult breeders in 2016-17.

Table S1. Summary of the discharge (flow) data sets used in this analysis. In particular, we summarize the flow conditions for each focal system used to compare flow conditions encountered by adult salmon during the 2013-14 winter compared to the longer-term record (40 years in most cases) and to the broader regional pattern (through the addition of flow data from 7 'other North Coast' rivers).

**Study system(s)**	**Representative USGS gage**	**Flow time series for analyses**
Fay and Tannery creeks (tributaries of the Salmon River), Sonoma County	Austin Creek NR Cazadero CA (tributary of Russian River) - USGS 11467200	10/1/2004 -09/30/2022 (n=19 years)
Russian River, Sonoma County	Russian R A Hacienda Bridge NR Guerneville CA – USGS 11467000	10/1/1982 -09/30/2022 (n=40 years)
South Fork Eel River, Mendocino County	SF Eel R NR Miranda CA - USGS 11476500	10/1/1982 -09/30/2022 (n=40 years)
Fox Creek (trib of the S. Fork Eel River)	Elder C NR Branscomb CA (tributary of the S. Fork Eel River) - USGS 11475560	10/1/1982 -09/30/2022 (n=40 years)
Mattole River, Humboldt/Mendocino County	Mattole R NR Petrolia CA - USGS 11469000	10/1/1982 -09/30/2022 (n=40 years)
Mendocino Coastal Streams, Mendocino County	Noyo R NR Fort Bragg CA - USGS 11468500	10/1/1982 -09/30/2022 (n=40 years)
'Other North Coast' rivers	Walker C NR Marshall CA – USGS 11460750	10/1/1983 -09/30/2022 (n=39 years)
'Other North Coast' rivers	Smith R NR Crescent City CA - USGS 11532500	10/1/1982 -09/30/2022 (n=40 years)
'Other North Coast' rivers	Klamath R NR Klamath CA – USGS 11530500	10/1/1982 -09/30/2022 (n=40 years)
'Other North Coast' rivers	Redwood C A Orick CA - USGS 11482500	10/1/1982 -09/30/2022 (n=40 years)
'Other North Coast' rivers	Little R NR Trinidad CA – USGS 11481200	10/1/1982 -09/30/2022 (n=40 years)
'Other North Coast' rivers	Mad R NR Arcata CA - USGS 11481000	10/1/1982 -09/30/2022 (n=40 years)
'Other North Coast' rivers	Van Duzen R NR Bridgeville CA - USGS 11478500	10/1/1982 -09/30/2022 (n=40 years)

Table S2 Summary of the biological data used in our analyses. In particular, we present a row for each system summarizing the data available to compare with data from the 2013-14 winter (adult observations) and/or 2014 summer (juvenile observations) seasons.

**System**	**Data source/agency**	**Species**	**Life stage monitored**	**Season surveyed**	**Years available to compare to 2013-14 data**
Fay Creek (tributary of the Salmon River), Sonoma County	UC-Berkeley	Coho salmon, Steelhead trout	Juveniles	Summer	4 summers: 2012, 2013, 2015, 2016
Tannery Creek (tributary of the Salmon River), Sonoma County	UC-Berkeley	Coho salmon, Steelhead trout	Juveniles	Summer	4 summers: 2012, 2013, 2015, 2016
Russian River, Sonoma County	California Sea Grant, Sonoma Water	Coho salmon	Adults	Winter	9 winters: 2012-13, 2014-15 to 2021-22
			Juveniles	Summer	8 summers: 2015 to 2022
South Fork Eel River, Mendocino County	CDFW	Coho salmon, Chinook salmon, Steelhead trout	Adults	Winter	9 winters: 2010-11 to 2012-13, 2014-15 to 2017-18, 2020-21 to 2021-22
Fox Creek (tributary of the South Fork Eel River)	UC-Berkeley	Steelhead trout	Juveniles	Summer	2 summers: 2015, 2016
Mattole River, Humboldt/Mendocino County	Mattole Salmon Group	Chinook salmon	Adults	Winter	11 winters: 2010-11 to 2012-13, 2014-15 to 2021-22
Noyo, Navarro, Pudding, Ten Mile rivers (within the Mendocino	CDFW	Coho salmon	Adults	Winter	14 winters: 2008-09 to 2012-13, 2014-15 to 2022-23

Coastal Streams), Mendocino County					
Caspar Creek (within the Mendocino Coastal Streams), Mendocino County	CDFW	Coho salmon	Adults	Winter	11 winters: 2008-09 to 2012-13, 2014-15 to 2019-20
Big, Garcia River, South Fork Albion River (within the Mendocino Coastal Streams), Mendocino County	CDFW	Coho salmon	Adults	Winter	13 winters: 2008-09 to 2012-13, 2014-15 to 2017-18, 2019-20 to 2022-23

Table S3. Summary of the impacts of the delayed rains during winter 2013-14 on three species of salmonid fishes in California, including shifts in their breeding phenology and location, as well as population impacts.

Location	Species	Life stages monitored	Delayed breeding	Shift in breeding location	Population impact
Salmon Creek[1], Sonoma County	Coho salmon	Juveniles	Unknown	Unknown	Cohort loss in one tributary
Russian River[2], Sonoma County	Coho salmon	Adults, juveniles	Yes	Reduced access to tributary breeding habitat	Contraction of juvenile rearing habitat, reduced juvenile production
South Fork Eel River[1,3], Mendocino County	Chinook salmon	Adults	Yes	Breeding in mainstem (considered uncommon in this system)	Unknown
	Coho salmon	Adults	Yes	Unknown	Unknown
	Steelhead trout	Adults	No	Unknown	Unknown
	Steelhead trout	Juveniles	Unknown	Reduced access to tributary breeding habitat	Cohort loss in one tributary (Fox Creek)
Mattole River[4], Humboldt County	Chinook salmon	Adults	yes	Concentrated in lowermost mainstem, due to a flow barrier at river mile 27	Unknown
Mendocino Coastal Streams[3], Mendocino County	Coho salmon	Adults	Unknown	Lost access to entire watersheds due to sand bar at mouth	Cohort failure at watershed scale (Pudding, Caspar, Navarro)

Footnotes correspond to data sources: [1]UC Berkeley, [2]California Sea Grant, [3]California Department of Fish and Wildlife, [4]Mattole Salmon Group

SI References

1. W. I. Atlas, W. J. Palen, Prey Vulnerability Limits Top-Down Control and Alters Reciprocal Feedbacks in a Subsidized Model Food Web. *PLoS ONE* **9**, e85830 (2014).

2. H. Uno, M. E. Power, Mainstem-tributary linkages by mayfly migration help sustain salmonids in a warming river network. *Ecology Letters* **18**, 1012–1020 (2015).

3. California Department of Fish and Wildlife and U.S. Army Corps of Engineers, Hatchery and genetics management plan: Don Clausen Fish Hatchery Russian River Coho Salmon Captive Broodstock Program. (2017).

4. CDFG, Recovery Strategy for California Coho Salmon. Report to the California Fish and Game Commission. 594 pp. (2004).

5. NMFS, Final recovery plan for Central California Coast coho salmon evolutionarily significant unit. (2012).

6. P. B. Adams, L. B. Boydstun, S. P. Gallagher, M. K. Lacy, T. McDonald, California Coastal Salmonid Population Monitoring: Strategy, Design, and Methods. *Fish Bulletin* **180** (2011).
